# Obesity, Inflammation, and Clinical Outcomes in COVID-19: A Multicenter Prospective Cohort Study

**DOI:** 10.1210/clinem/dgae273

**Published:** 2024-04-18

**Authors:** Christina G Hutten, Kishan Padalia, Alexi Vasbinder, Yiyuan Huang, Anis Ismail, Ian Pizzo, Kristen Machado Diaz, Tonimarie Catalan, Feriel Presswalla, Elizabeth Anderson, Grace Erne, Brayden Bitterman, Pennelope Blakely, Evangelos J Giamarellos-Bourboulis, Sven H Loosen, Frank Tacke, Athanasios Chalkias, Jochen Reiser, Jesper Eugen-Olsen, Mousumi Banerjee, Rodica Pop-Busui, Salim S Hayek

**Affiliations:** Division of Cardiology, Department of Internal Medicine, University of Michigan, Ann Arbor MI 48109, USA; Department of Internal Medicine, University of Michigan, Ann Arbor MI 48109, USA; Division of Cardiology, Department of Internal Medicine, University of Michigan, Ann Arbor MI 48109, USA; Department of Biostatistics, School of Public Health, University of Michigan, Ann Arbor, MI 48109, USA; Division of Cardiology, Department of Internal Medicine, University of Michigan, Ann Arbor MI 48109, USA; Division of Cardiology, Department of Internal Medicine, University of Michigan, Ann Arbor MI 48109, USA; Division of Cardiology, Department of Internal Medicine, University of Michigan, Ann Arbor MI 48109, USA; Division of Cardiology, Department of Internal Medicine, University of Michigan, Ann Arbor MI 48109, USA; Division of Cardiology, Department of Internal Medicine, University of Michigan, Ann Arbor MI 48109, USA; Division of Cardiology, Department of Internal Medicine, University of Michigan, Ann Arbor MI 48109, USA; Division of Cardiology, Department of Internal Medicine, University of Michigan, Ann Arbor MI 48109, USA; Division of Cardiology, Department of Internal Medicine, University of Michigan, Ann Arbor MI 48109, USA; Division of Cardiology, Department of Internal Medicine, University of Michigan, Ann Arbor MI 48109, USA; 4th Department of Internal Medicine, National and Kapodistrian University of Athens, Medical School, 124 62 Athens, Greece; Clinic for Gastroenterology, Hepatology and Infectious Diseases, Medical Faculty, University Hospital Düsseldorf, 40225 Düsseldorf, Germany; Department of Hepatology and Gastroenterology, Charité—Universitätsmedizin Berlin, 13353 Berlin, Germany; Institute for Translational Medicine and Therapeutics, University of Pennsylvania Perelman School of Medicine, Philadelphia, PA 19104, USA; Outcomes Research Consortium, Cleveland, OH 44195, USA; Department of Medicine, Rush University Medical Center, Chicago, IL 60612, USA; Department of Clinical Research, Copenhagen University Hospital Amager and Hvidovre, 2650 Hvidovre, Denmark; Department of Biostatistics, School of Public Health, University of Michigan, Ann Arbor, MI 48109, USA; Division of Metabolism, Endocrinology and Diabetes, Department of Internal Medicine, University of Michigan, Ann Arbor, MI 48109, USA; Division of Cardiology, Department of Internal Medicine, University of Michigan, Ann Arbor MI 48109, USA

**Keywords:** COVID-19, SARS-CoV-2, coronavirus, obesity, BMI, inflammation, biomarkers

## Abstract

**Context:**

Obesity is a risk factor for coronavirus disease 2019 (COVID-19)-related outcomes; however, the mechanism remains unclear.

**Objective:**

The objective of this analysis was to determine whether inflammation mediates the association between obesity and COVID-19 outcomes.

**Methods:**

The International Study of Inflammation in COVID-19 (ISIC): A Prospective Multi-Center Observational Study Examining the Role of Biomarkers of Inflammation in Predicting Covid-19 Related Outcomes in Hospitalized Patients, was conducted at 10 hospitals in the United States and Europe. Participants were adults hospitalized specifically for COVID-19 between February 1, 2020, through October 19, 2022. Inflammatory biomarkers, including soluble urokinase plasminogen activator receptor (suPAR), were measured at admission. Associations were examined between body mass index (BMI, kg/m^2^) and a composite of death, need for mechanical ventilation, and renal replacement therapy, stratified by pre- and post-Omicron variants. The contribution of inflammation to the relationship between obesity and outcomes was assessed.

**Results:**

Among 4644 participants (mean age 59.3, 45.6% male, 21.8% BMI ≥ 35), those with BMI > 40 (n = 485) had 55% higher odds of the composite outcome (95% CI, 1.21-1.98) compared with nonobese individuals (BMI < 30, n = 2358) in multivariable analysis. In multiple mediation analysis, only suPAR remained a significant mediator between BMI and composite outcome. Associations were amplified for participants younger than 65 years and with pre-Omicron variants.

**Conclusion:**

Obesity is associated with worse outcomes in COVID-19, notably in younger participants and in the pre-Omicron era. Inflammation, as measured by suPAR, is a significant mediator of the association between obesity and COVID-19 outcomes.

Severe acute respiratory syndrome coronavirus 2 (SARS-CoV-2) is responsible for the most devastating pandemic in generations, causing hundreds of millions of coronavirus disease 2019 (COVID-19) cases and tens of millions of deaths worldwide (1). Severe COVID-19 is a systemic inflammatory syndrome triggered by SARS-CoV-2 infection and characterized by immune cell dysfunction and elevated levels of cytokines leading to multiorgan failure (2). This persistent hyperinflammatory response to SARS-CoV-2 infection, often termed *cytokine storm*, is associated with high levels of thrombo-inflammatory biomarkers, including C-reactive protein (CRP), ferritin, D-dimer, interleukin-6 (IL-6), procalcitonin, and soluble urokinase plasminogen activator receptor (suPAR) (3).

Obesity, resulting from increased adipose tissue leading to endocrine and metabolic dysregulation, has been identified as a risk factor for COVID-19 infection and outcomes (4-6). Obesity promotes a chronic inflammatory state characterized by abnormal activation of leukocytes that infiltrate adipocytes and increase the secretion of proinflammatory cytokines (7-10). This proinflammatory milieu in patients with obesity is thought to increase the risk of severe COVID-19 by causing lymphocyte dysfunction (11), complement-mediated endothelial dysfunction (12), modulation of the angiotensin-converting enzyme 2 receptor expression (13), and changes in lung mechanics and pulmonary function (14), among other processes.

Whether the association between obesity and worse outcomes in COVID-19 is mediated through a heightened inflammatory response remains unclear. The relationship between obesity and inflammation in COVID-19 in studies to date have produced inconsistent findings due to significant limitations. With few exceptions, these studies were conducted at a single center with a small sample size, retrospective in nature, limited to observations early in the pandemic and use of conventional biomarkers, or focused on select populations such as patients in the intensive care unit or those defined solely by a positive SARS-CoV-2 test (15-17).

The objective of this study is to characterize the link between obesity, inflammation, and outcomes in COVID-19. We leveraged the International Study of Inflammation in COVID-19 (ISIC), a prospective multicenter study of patients hospitalized specifically for COVID-19 and in whom conventional as well as novel thrombo-inflammatory biomarker levels were measured at the time of admission.

## Methods

### Study Cohort

The ISIC is a multi-national observational prospective cohort study designed to advance the understanding of the role of inflammation in COVID-19 (18-22). Participating centers include University of Michigan in Ann Arbor, Michigan, USA; Rush University in Chicago, Illinois, USA; Copenhagen University Hospital Hvidovre, Denmark; Attikon University Hospital in Athens, Greece; Charité University Medicine Berlin, Germany; University Hospital of Dusseldorf, Germany; University of Thessaly, Greece; University Hospital of Cologne, Germany; and Medical University of Graz, Austria (Supplementary Table S1 (23)).

For this substudy of the ISIC, inclusion criteria were: (i) adults (≥ 18 years old) hospitalized for symptomatic COVID-19; (ii) SARS-CoV-2 infection confirmed with a positive reverse transcriptase polymerase chain reaction test of a nasopharyngeal or oropharyngeal sample; (iii) at least one blood sample collected within 48 hours of admission for biomarker testing; (iv) admission height and weight data available to calculate body mass index (BMI); and (v) admitted February 1, 2020 through October 19, 2022. Patients with SARS-CoV-2 infection not admitted primarily for symptomatic COVID-19 were excluded.

All sites were approved by an ethical committee for conduct, collected patient consent, or received a waiver of consent. Institutional or ethical review board approvals were obtained at each clinical site per local and/or institutional policies: University of Michigan Medical School IRBMED (HUM00178971), The Ethical Committee of the University Hospital of Larisa (17543), Ethics Committee of the Charité- Universitätsmedizin Berlin (EA2/066/20-Pa-COVID), Ethics Committee at the Faculty of Medicine of Heinrich Heine University Dusseldorf (5350), Athens Ethics Committee (IS 021-20), Danish Patient Safety Authority (31-1521-319), the Capital Region of Denmark (R-20064514; data record P-2020-513). Patient written informed consent was obtained for participants or by a healthcare proxy unless a waiver of consent was approved (Michigan, Larisa, and Denmark sites). The ISIC was registered with ClinicalTrials.gov (NCT04818866). An MTA was created between all institutions for sharing data with the University of Michigan.

### Patient and Public Involvement

The multicenter study design and the need to generate data quickly during the pandemic precluded patient or public involvement in the design or conduct of the study.

### Study Definitions

The data collected included details regarding clinical presentation, medical history, home medications, laboratory values, imaging studies, inpatient treatment, hospital course, and in-hospital outcomes. Data were extracted from electronic medical records through manual chart review by at least 2 reviewers per site and entered in an online repository managed with Research Electronic Data Capture hosted at the University of Michigan.

BMI was calculated using weight and height on admission with the following equation: BMI = weight (kg)/[height (m)]^2^. Patients were categorized into 4 groups by BMI: BMI < 30.0 kg/m^2^ was classified as nonobese, 30.0 to 34.9 kg/m^2^ as class I obesity, 35.0 to 39.9 kg/m^2^ as class II obesity, and ≥ 40 kg/m^2^ as class III obesity (24).

Patients were followed until in-hospital death or hospital discharge. The primary outcome was a composite of in-hospital death, mechanical ventilation, and renal replacement therapy (RRT).

### Biomarker Measurements

Biomarkers measured included CRP, ferritin, D-dimer, procalcitonin, IL-6, and suPAR. The first biomarker level obtained within 48 hours of admission was used for this analysis. Levels of CRP (mg/dL), ferritin (ng/mL), D-dimer (mg/L fibrinogen equivalent units [FEU]), and procalcitonin (ng/mL) were measured by the central laboratory at each of the respective sites of enrollment. SuPAR was measured using a commercially available enzyme-linked immunosorbent assay with a double monoclonal antibody sandwich (Virogates Cat# E001, RRID:AB_3095558). Per the assay manufacturer, kits have a lower detection limit of 100 pg/mL and intra- and inter-assay variation of 2.75% and 9.17%, respectively. Interleukin-6 was measured (Ortho Clinical Diagnostics Cat# 6199971, RRID:AB_3095595), with an assay range of 3.82 to 1680 pg/mL, limit of detection of 0.95 pg/mL, limit of quantification of 1.04 pg/mL and inter- and intra-assay variation of < 6%.

### Statistical Analysis

The summary statistics of demographics, clinical characteristics, and median levels of relevant biomarkers were stratified by predefined BMI groups: nonobese, class I obesity, class II obesity, and class III obesity. Categorical variables were presented as the total frequency (n) and percentage (%). Continuous variables were presented as mean and SD for normally distributed variables or median and 25th to 75th interquartile range (IQR) for non-normally distributed variables. Differences were evaluated using the Chi-squared test or pairwise Wilcoxon rank sum tests for categorical variables and either the Kruskal-Wallis test or one-way analysis of variance for continuous variables.

#### Relationship between BMI and biomarkers

Spearman's rank was applied to test for correlations between BMI as a continuous variable and each inflammatory biomarker. These relationships were further investigated using multivariable linear regressions for the association between continuous BMI as the dependent variable and each log_2_-transformed inflammatory biomarker (interpreted as per 100% increase for every 1 unit increase in BMI), in separate models, adjusting for clinical covariates determined a priori: age, sex, race, history of smoking, creatinine-derived estimated glomerular filtration rate (eGFR) on admission, hypertension, diabetes mellitus, coronary artery disease (CAD), and congestive heart failure (CHF). Beta coefficients and 95% CI were calculated.

#### BMI and outcomes

Multivariable logistic regressions were performed to test for the associations between BMI and the composite outcome and each outcome separately (in-hospital death, need for mechanical ventilation, and RRT). For the purpose of our analysis, any occurrence of in-hospital death, the requirement for mechanical ventilation, or RRT was recorded as meeting the composite outcome criterion. This approach was adopted to ensure that each participant was accounted for only once in the composite outcome, regardless of experiencing multiple adverse events. BMI was evaluated as both a continuous variable (per each 5 kg/m^2^) and a categorical variable by the predefined categories. Models were adjusted for age, sex, race, diabetes mellitus, hypertension, CAD, CHF, smoking history, and eGFR at admission. Odds ratios (OR) and 95% CIs were determined. The association between BMI categories and the composite outcome was explored utilizing a stepwise approach: Model 1 included demographic characteristics (age, sex, and race); Model 2 included clinical characteristics (diabetes mellitus, hypertension, CAD, CHF, smoking history, and eGFR at admission); and Model 3 included inflammatory biomarker, suPAR or CRP.

We also investigated the possibility of effect modification due to differences in baseline characteristics by evaluating the interaction between binary BMI (≥ 35 vs < 35 kg/m^2^) and relevant subgroups. Logistic regressions were repeated for the association of the binary BMI and the composite outcome stratified by subgroups. The subgroups evaluated included: age with cutoff of 65 years, sex, race, diabetes mellitus, hypertension, CAD, CHF, smoking history, eGFR on admission with cutoff 60 mL/min/1.73m^2^, continent of enrollment, and variant type (pre-Omicron vs Omicron based on pre- or post-admission date of 10/31/2021). We selected this cutoff date based on the World Health Organization's identification of the Omicron variant's emergence in November 2021. By the end of that month, Omicron had become the predominant strain globally (25). *P* values for the interaction were calculated.

#### Mediation analysis

Mediation analysis was conducted to evaluate the proportion of the effect of binary BMI (≥ 35 and < 35 kg/m^2^) on the composite outcome mediated by each inflammatory biomarker (26, 27). Analyses were conducted for each log_2_-transformed biomarker using complete cases (28). Logistics regressions were used to assess the total effect of BMI on the composite outcome, adjusting for age, sex, race, history of smoking, eGFR at admission, hypertension, diabetes, CAD, CHF, and each inflammatory biomarker separately. Linear regressions were applied for the mediator models of the biomarkers on BMI, adjusting for the same set of covariates as the total effect model. Mediation analysis was performed using the “mediation” package in R, where the proportion mediated was calculated using the ratio of the indirect effect to the total effect. We stratified the results by BMI categories (above and below 35 kg/m^2^) to assess how these mediating effects might vary across different obesity levels, with results further stratified by age using a cutoff of 65 years. Multiple mediation analysis was performed using the “mma” package in R adjusting for age, sex, race, history of smoking, eGFR at admission, hypertension, diabetes, CAD, CHF and suPAR with CRP, or suPAR with IL-6.

Complete case analyses were performed for all regression modeling. All analyses were performed using R Version 4.2.2 (R Foundation for Statistical Computing, Vienna, Austria). A two-sided *P* value < .05 was used to determine statistical significance.

## Results

### Cohort Characteristics

A total of 4464 adult patients hospitalized for symptomatic COVID-19 were included in this analysis. Of these, 52.8% were categorized as nonobese, 25.3% as class I obesity, 11.0% as class II obesity, and 10.9% as class III obesity (Table 1). There were several notable differences in baseline characteristics across BMI groups. Patients with higher BMI were more likely to be Black/African American and have a higher prevalence of several comorbidities, including diabetes mellitus, hypertension, asthma, and obstructive sleep apnea (Table 1). Crucially, a significant difference in mean age was noted among the BMI groups, with the nonobese group being the oldest. This underscores the potential confounding influence of age on the association between BMI and clinical outcomes in COVID-19.

**Table 1.** Summary statistics of baseline characteristics by BMI categories

	Nonobese (< 30 kg/m^2^)	Class I obesity (30-34.9 kg/m^2^)	Class II obesity (35-39.9 kg/m^2^)	Class III obesity (≥ 40 kg/m^2^)	*P* value
	N = 2358, 52.8%	N = 1131, 25.3%	N = 490, 11.0%	N = 485, 10.9%	
**Demographics**					
Female, n (%)	938 (39.8%)	541 (47.8%)	261 (53.3%)	296 (60.9%)	<.001
Age, years, mean (SD)	63.6 (17.7)	56.3 (15.6)	55.1 (15.2)	49.7 (14.9)	<.001
BMI, kg/m^2^, mean (SD)	25.7 (3.2)	32.1 (1.4)	37.1 (1.4)	48.3 (28.2)	<.001
Black race, n (%)	292 (12.4%)	155 (13.7%)	97 (19.8%)	149 (30.7%)	<.001
**Comorbidities, n (%)**					
Diabetes mellitus	557 (23.6%)	338 (29.9%)	208 (42.4%)	218 (44.9%)	<.001
Hypertension	1060 (45.0%)	533 (47.1%)	297 (60.6%)	294 (60.5%)	<.001
Coronary artery disease	368 (15.6%)	150 (13.3%)	63 (12.9%)	36 (7.4%)	<.001
Congestive heart failure	235 (10.0%)	82 (7.3%)	64 (13.1%)	57 (11.7%)	.001
Asthma	230 (9.8%)	139 (12.3%)	92 (18.8%)	120 (24.7%)	<.001
Apnea	202 (8.6%)	159 (14.1%)	114 (23.3%)	173 (35.6%)	<.001
Current or former smoking	1020 (43.3%)	453 (40.1%)	208 (42.4%)	168 (34.6%)	.022
**Biomarker levels, median (IQR)**					
CRP (mg/dL)	6.3 (2.5-12.4)	6.2 (2.5-12.9)	7.3 (3.1-15.3)	7.0 (3.3-13.2)	.032
Ferritin (ng/mL)	503 (203-1053)	518 (234-1055)	598 (240-1091)	396 (151-913)	<.001
D-dimer (mg/L FEU)	0.85 (0.45-1.89)	0.72 (0.41-1.45)	0.83 (0.49-1.70)	0.93 (0.52-2.21)	<.001
IL-6 (pg/mL)	36.1 (12.5-86.9)	33.8 (12.5-74.9)	34.0 (12.5-84.1)	28.2 (12.5-77.5)	.5
suPAR (ng/mL)	5.9 (4.1-9.0)	5.8 (4.1-8.8)	6.7 (4.7-9.2)	6.7(4.6-9.5)	<.001
Procalcitonin (ng/mL)	0.13 (0.07-0.36)	0.12 (0.07-0.27)	0.13 (0.08-0.30)	0.13 (0.07-0.36)	.1
**Outcomes**					
Composite outcome, n (%)	556 (23.7%)	235 (20.8%)	131 (26.7%)	139 (28.8%)	.003
Mortality, n (%)	324 (13.7%)	116 (10.3%)	52 (10.6%)	48 (9.9%)	.005
Mechanical ventilation, n (%)	410 (17.4%)	202 (17.9%)	116 (23.7%)	126 (25.9%)	<.001
Renal replacement therapy, n (%)	147 (6.2%)	62 (5.5%)	48 (9.8%)	56 (11.5%)	<.001

Abbreviations: BMI, body mass index; CRP, C-reactive protein; FEU, fibrinogen equivalent units; IL-6, interleukin 6; IQR, interquartile range; suPAR, soluble urokinase plasminogen activator receptor.

### BMI and Outcomes

**Figure 1.**
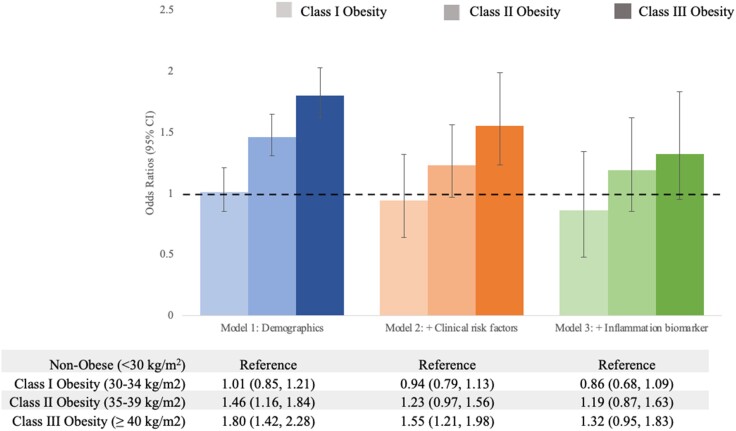
Odds ratios for composite outcome by BMI categories. Bar graphs showing the odds ratios and 95% CI for the association of obesity with the composite outcome using nonobese (< 30 kg/m^2^) as reference vs Class I obese (30-34.9 kg/m^2^), Class II obese (35-39.9 kg/m^2^), and Class III obese (≥ 40 kg/m^2^) patients. Model 1 (blue): adjusted for age, sex, and race; Model 2 (orange): adjusted for Model 1 covariates plus history of smoking, eGFR at admission, hypertension, diabetes, coronary artery disease, and congestive heart failure; Model 3 (green): adjusted for Model 2 covariates plus suPAR. Abbreviations: BMI, body mass index; eGFR, estimated glomerular filtration rate; suPAR, soluble urokinase plasminogen activator receptor.

Overall, there were 1061 (23.8%) participants who met the composite outcome, 540 (12.1%) deaths, 854 (19.1%) patients who required mechanical ventilation, and 313 (7.0%) who required RRT. In unadjusted analyses, mortality appeared to be higher in the nonobese category (BMI < 30 kg/m^2^) compared with higher BMI categories (Table 1). In multivariable analyses, per each 5 kg/m^2^ increment in BMI, the ORs of the composite outcome, mortality, needing mechanical ventilation, or needing RRT were 1.12 (95% CI, 1.06 to 1.17), 1.07 (95% CI, 1.00 to 1.14), 1.14 (95% CI, 1.09 to 1.20), and 1.20 (95% CI, 1.11 to 1.29), respectively. When obesity was treated as a categorical variable, participants with class II and class III obesity were more likely to experience the composite outcome compared to nonobese as reference after adjusting for demographic characteristics (Model 1, Fig. 1). Further adjustment for clinical risk factors (Model 2) and inflammation as measured by suPAR (Model 3) dampened the association between categorized obesity and the composite outcome (Fig. 1). Adjustment of model 3 by CRP instead of suPAR revealed a similar dampening of the association between categorized obesity and the composite outcome (class III obesity vs nonobese reference, adjusted OR [aOR] 1.18 [95% CI, 0.88-1.59]). Results were unchanged after adjusting for institution (aOR 1.56 [95% CI, 1.30-1.88]). When outcomes were examined individually, patients with class II and class III obesity had a higher likelihood for need for mechanical ventilation or need for RRT, but not mortality (Table 2). BMI was not associated with mortality when examining BMI as a categorical or continuous variable in adjusted analyses.

**Table 2.** Binary logistic regression results of clinical outcomes by BMI group

BMI group(kg/m^2^)	Composite outcome	Mortality	Mechanical ventilation	Renal replacement therapy
	OR (95% CI)	OR (95% CI)	OR (95% CI)	OR (95% CI)
Nonobese (< 30)	Ref	Ref	Ref	Ref
Class I (30-34.9)	0.94 (0.79, 1.13)	0.97 (0.77, 1.24)	1.05 (0.86, 1.27)	0.96 (0.69, 1.33)
Class II (35-39.9)	1.23 (0.97, 1.56)	1.02 (0.73, 1.42)	**1.38 (1.08, 1.77)**	**1.51 (1.04, 2.18)**
Class III (≥ 40)	**1.55 (1.21, 1.98)**	1.24 (0.87, 1.78)	**1.64 (1.27, 2.12)**	**2.01 (1.38, 2.93)**

Note: models are adjusted for age, sex, race, history of smoking, admission eGFR, hypertension, diabetes, coronary artery disease, and congestive heart failure. Bolded values are statistically significant (*P* < .05).

Abbreviations: BMI, body mass index; OR, odds ratio.

#### Sensitivity analyses

**Figure 2.**
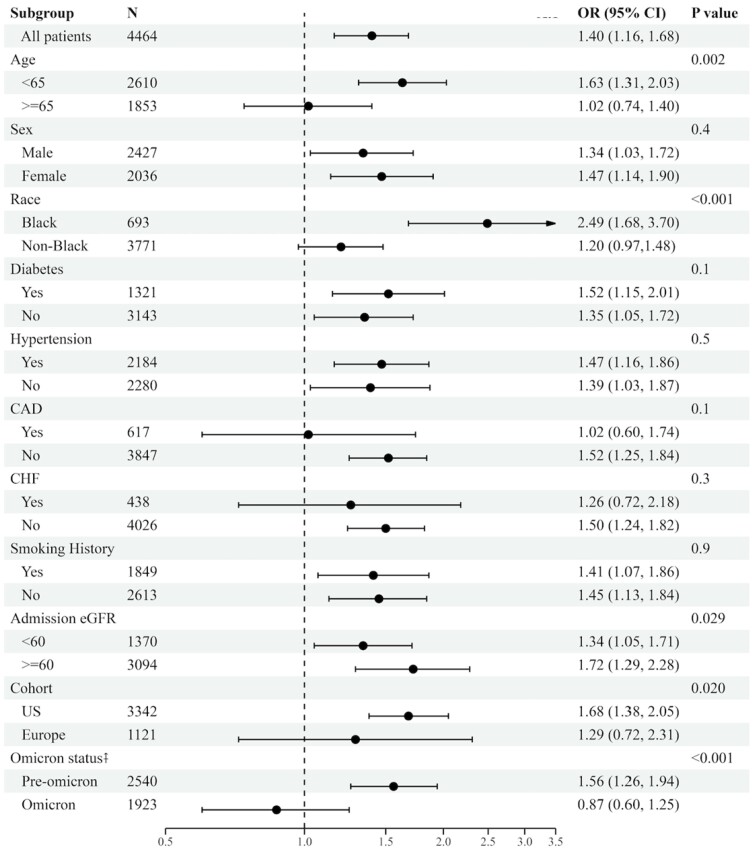
Association of BMI with the composite outcome across subgroups. Forest plot showing the risk of composite outcome (mortality, need for mechanical ventilation, need for renal replacement therapy) for BMI > 35 kg/m^2^ compared to < 35 kg/m^2^ in specified subgroups. *P* value is for the interaction between BMI and each subgroup. The model with all patients is adjusted for age, sex, history of smoking, estimated GFR at admission, hypertension, diabetes, coronary artery disease, and coronary heart failure. All stratified models are adjusted by the same covariates, minus the stratification variable. Abbreviations: BMI, body mass index; CAD, coronary artery disease; CHF, congestive heart failure; eGFR, estimated glomerular filtration rate; OR, odds ratio.

**Figure 3.**
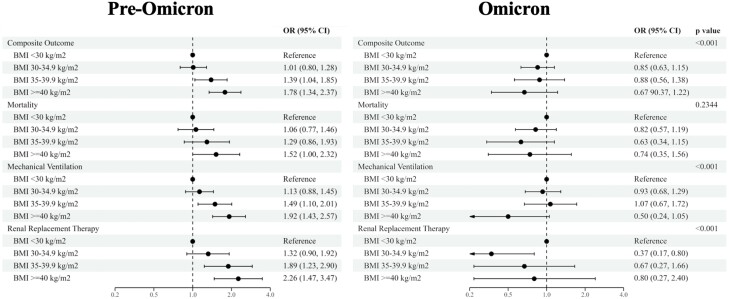
Associations of BMI categories with COVID-19 outcomes stratified by variant. Forest plot showing the risk of composite outcome, mortality, intubation, and dialysis by body mass index (BMI) categories, nonobese (< 30 kg/m^2^), Class I obese (30-34.9 kg/m^2^), Class II obese (35-39.9 kg/m^2^), and Class III obese (≥ 40 kg/m^2^) and stratified by Omicron status, defined as admission date before or after October 31, 2021. All models were adjusted for age, sex, history of smoking, estimated GFR at admission, hypertension, diabetes, coronary artery disease, and coronary heart failure.

To better understand divergences in the association between BMI and outcomes, we explored interactions between BMI and relevant clinical characteristics. We found significant differences in the effect of BMI (≥ 35 vs < 35 kg/m^2^) on the composite outcome between groups of age (*P* = .003), race (*P* < .001), eGFR at admission (*P* = .029), cohort (US or Europe) (*P* = .020), and type of variant (pre- or post-Omicron) (*P* < .001) (Fig. 2). Those with BMI ≥ 35 kg/m^2^ who exhibited the composite outcome were more likely to be under 65 years of age, be Black/African American, have ≥ 60 eGFR at admission, and be in the US cohort when adjusted for all other covariates. When stratifying by variant, BMI was associated with outcomes only in patients with the pre-Omicron variant. Furthermore, when BMI categories (nonobese, class I obesity, class II obesity, and class III obesity) were stratified by pre- and post-Omicron variant, the associations with each outcome showed higher odds with increasing severity of obesity pre-Omicron; however, the association of BMI category with each outcome no longer showed discernable associations post-Omicron (Fig. 3).

### BMI and Inflammatory Biomarkers

**Figure 4.**
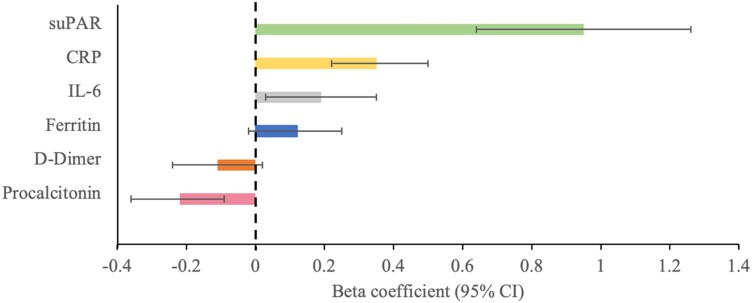
Beta coefficients for inflammatory biomarkers predicting BMI. Bar graph showing the beta coefficients and 95% CI for the association between inflammatory biomarkers (suPAR, CRP, IL-6, ferritin, D-Dimer, procalcitonin) and body mass index (BMI). Models are adjusted for age, sex, race, history of smoking, admission eGFR, hypertension, diabetes, coronary artery disease, and congestive heart failure. Abbreviations: CRP, C-reactive protein; eGFR, estimated glomerular filtration rate; IL-6, interleukin-6; suPAR, soluble urokinase plasminogen activator receptor.

Levels of CRP, ferritin, D-dimer, and suPAR differed significantly by BMI categories (Table 1). In unadjusted analyses, BMI as a continuous variable correlated positively with suPAR (*r* = 0.06; *P* = .002) and CRP (*r* = 0.04; *P* = .018) but not with ferritin (*r* = 0.01; *P* = .8), D-dimer (*r* = −0.01; *P* = .5), IL-6 (*r* = 0.01, *P* = .8), or procalcitonin (*r* = −0.03, *P* = .1). In multivariable linear regression analyses, CRP, IL-6, and suPAR were associated with higher BMI while procalcitonin was associated with lower BMI (Fig. 4).

Given the confounding influence of age on the association between BMI and outcomes, we examined whether levels of inflammatory markers differed between obese and nonobese group of patients stratified by age. Among measured biomarkers, we found only suPAR levels to differ significantly according to age group (*P* interaction = .005). In patients age < 65 years, the median difference in suPAR levels between obese and nonobese patients was 1.5 ng/mL. Conversely, in patients age ≥ 65, the difference was only 0.8 ng/mL.

### Mediation Analysis

We conducted a mediation analysis to assess the contribution of inflammatory biomarkers to the association between obesity and the composite outcome. When assessing for multiple mediation effects, the proportion of suPAR and CRP together was 51.2% (*P* < .001); however, only the estimate for the indirect effect of suPAR was significant (*P* < .001). Similarly, when suPAR and IL-6 were assessed in joint mediation, the proportion together was 23.6% (*P* < .001) and only the estimate for the indirect effect of suPAR remained significant (*P* < .001) (Supplementary Table S2 (23)). We observed that suPAR exhibited a more pronounced additive effect on the association between BMI and COVID-19 in-hospitalization outcomes compared with CRP and IL-6. Importantly, we acknowledge the possibility of interconnected mediating pathways, where the effects of CRP and IL-6 on the outcomes could be partially mediated through suPAR or vice versa. This complexity highlights the intricate interplay among these biomarkers in the inflammatory response to COVID-19. Stratified mediation analysis by age (≥ 65 vs < 65 years) showed that inflammation plays a larger role in younger patients than older patients (proportion mediated through suPAR in patients age < 65 years is 53.6% (*P* < .001) vs 29.0% (*P* = .7) for patients ≥ 65 years old, respectively).

## Discussion

In this multi-national observational cohort study, we sought to elucidate the relationship between obesity, inflammation, and clinical outcomes in patients hospitalized with COVID-19. Our findings indicate that obesity, particularly class II and III obesity (BMI ≥ 35 kg/m²), is associated with an increased risk of adverse outcomes, including the need for mechanical ventilation and RRT. Notably, this association was more pronounced in Black, younger patients, and during the pre-Omicron era of the pandemic. The role of inflammation as a mediator in the relationship between obesity and COVID-19 outcomes was a focal point of our investigation. Among several biomarkers of inflammation elevated in COVID-19, suPAR emerged as the predominant mediator of the association between obesity and outcomes. This suggests that suPAR may play a more central role in the inflammatory response among obese COVID-19 patients, offering novel mechanistic insights. Differences in the role of inflammation between the pre-Omicron and the Omicron era, underscore the evolving mechanisms related to COVID-19 outcomes over the course of the pandemic, emphasizing the need for continuous research adaptation and exploration.

Our study corroborates previous research linking severe obesity to poorer COVID-19 related outcomes (5, 29), and extends these findings by highlighting the impact of inflammation and delineating the association's dependency on factors like the predominant SARS-CoV-2 variant and patient age. Most existing studies focus on data from the pre-Omicron era, characterized by more severe disease outcomes (30). Our analysis reveals a significant association between obesity and COVID-19 outcomes in the pre-Omicron era, which diminishes with the emergence of the Omicron variant. This could be attributed to the altered virulence of initial SARS-CoV-2 strains, such as Delta, and the protective effects of vaccination.

We found that obesity was more strongly associated with outcomes in younger patients compared to older patients. This age-related divergence in the association between obesity and COVID-19 was initially reported by Kass et al (31), and may reflect the “obesity paradox” observed in other conditions such as diabetes, cardiovascular disease, and acute respiratory distress syndrome where obesity appears protective against poor outcomes (32-34). In the elderly, higher weight might indicate a less catabolic state, often contrasted with advanced chronic diseases associated with inflammation, cachexia, and lower body weight (35). Supporting this, our data show that younger obese patients had significantly elevated suPAR levels compared to their nonobese counterparts, a difference that was less pronounced in older patients. Inflammation, as indicated by suPAR levels, was a more substantial mediator in younger patients, suggesting a lesser role for obesity in driving chronic inflammation in the elderly.

We observed a pronounced disparity in the odds of the composite outcome between Black and non-Black participants, a finding that merits deeper examination. The differential impact of COVID-19 on these groups may stem from a confluence of factors, including but not limited to chronic inflammation, comorbidities, socio-economic conditions, genetic predispositions, and psychosocial stressors. These factors, individually or in combination, might influence the degree of inflammatory response to COVID-19, potentially exacerbated by the presence of obesity. Notably, in the Black population, the interplay between obesity and these factors may amplify the inflammatory response to COVID-19 infection, leading to more severe outcomes. Markers of social determinants of health were not included in our dataset, which constitutes a limitation of our study. The integration of these markers in future research could provide crucial insights into the mechanisms driving the disparities in inflammation, obesity, and COVID-19 outcomes, particularly among diverse populations.

Chronic inflammation encompasses a range of complex and distinct processes. The ISIC has aimed to quantify various aspects of thrombo-inflammation by measuring conventional biomarkers such as CRP, ferritin, D-dimer, and procalcitonin, as well as novel biomarkers like IL-6 and suPAR. Among these, suPAR emerged as the most strongly associated with obesity and the only significant mediator in the linkage between obesity and COVID-19 outcomes. SuPAR, an immune-derived signaling glycoprotein produced by myeloid cells, plays a role in the pathogenesis of kidney and cardiovascular diseases and, potentially, severe COVID-19 outcomes (21, 36, 37). Unlike acute phase reactants such as CRP or IL-6, suPAR levels, reflecting chronic innate immune activity, are generally stable and are influenced by limited factors, including smoking and RNA viruses like SARS-CoV-2 and HIV.

SuPAR has demonstrated robust predictive value for COVID-19-related outcomes, surpassing clinical risk models and conventional biomarkers (38, 39). Interestingly, an ISIC study found that suPAR accounted for most of the risk associated with diabetes mellitus in COVID-19 patients (18). COVID-19 is notable for causing dramatic increases in suPAR levels, potentially explaining its specific and strong correlation with disease severity. The production of suPAR by adipocytes may elucidate the higher levels observed in obesity (40). The exact mechanism by which suPAR contributes to respiratory failure in COVID-19 remains unclear. Recent findings suggest a direct interaction between suPAR and the SARS-CoV-2 spike protein, leading to glomerulopathy (41). Investigating whether similar interactions occur in other cell types could further elucidate the pathophysiology underlying the association between obesity and severe COVID-19 outcomes.

## Strengths and Limitations

The major strengths of our study include its large sample size and its prospective nature, in which patients who tested positive for SARS-CoV-2 were manually reviewed, and only patients hospitalized specifically for COVID-19 were included. Data was gathered by manual chart review rather than having relied on billing codes. Blood samples were systematically collected, and biomarkers measured on admission, reflecting severity of disease and minimizing confounding due to interventions. The Michigan Medicine Covid-19 Cohort (M^2^C^2^) and the University of Hvidovre cohorts—the largest 2 contributors to ISIC, enrolled patients consecutively. Other sites however did not consecutively enroll patients, leading to varying sample sizes and a risk of selection bias. Other limitations include the observational nature of the study, which precludes causal inferences and the potential for residual confounding.

## Conclusion

In summary, our study offers compelling evidence that obesity, particularly in younger patients and those infected with pre-Omicron variants, is associated with worse COVID-19 outcomes, mediated in part by inflammation as indicated by suPAR levels. These findings enrich the growing body of literature on the impact of obesity in infectious diseases and shed light on potential biomarkers for clinical decision-making in COVID-19 management. Future research should aim to unravel the mechanisms underlying the “obesity paradox” and explore the therapeutic potential of targeting suPAR-mediated pathways in the treatment of COVID-19.

## Data Availability

The data that support the findings of this study are available from the corresponding author upon reasonable request. Study protocol, statistical code, and data set summary data are available upon request after publication through a collaborative process. Data sets can be accessed upon approval of a submitted research proposal. Please contact penegonz@med.umich.edu for additional information.

## Trial Registration

ClinicalTrials.gov identifier: NCT04818866.

## Abbreviations

BMI: body mass index
CAD: coronary artery disease
CHF: congestive heart failure
CRP: C-reactive protein
eGFR: estimated glomerular filtration rate
IL-6: interleukin 6
ISIC: International Study of Inflammation in COVID-19
OR: odds ratio
RRT: renal replacement therapy
suPAR: soluble urokinase plasminogen activator receptor
